# Two Cases of Omental Torsion Mimicking Acute Appendicitis

**Published:** 2014-04-01

**Authors:** Feeroz Alam Khan, Naeem Liaqat, Sajid Hameed Dar, Asif Iqbal Sandhu, Sajid Nayyer

**Affiliations:** Department of Pediatric Surgery, Services Institute of Medical Sciences Lahore, Pakistan

**Keywords:** Omental torsion, Omentectomy, Appendectomy

## Abstract

Acute appendicitis is often simulated by other entities like mesenteric adenitis, worm infestation, Meckel’s diverticulitis, urinary tract infection and rarely omental torsion. We report two cases, a 6 year old boy and an 11 year old girl, who presented with symptoms and signs of acute appendicitis but upon exploration turned out to be omental torsion.

## INTRODUCTION

Omental torsion is a rare cause of acute abdominal pain. It often mimics acute appendicitis preoperatively.[1-5] We present two such cases in which a pre-operative diagnosis of acute appendicitis turned out to be primary omental torsion at surgery.

## CASE REPORT

**Case 1**

A 6-year-old boy presented with pain in right iliac fossa for one day. Pain was continuous in nature, severe in intensity with no aggravating or relieving factor and no shifting or radiation. On examination, a30 kg child, with pulse of 124 beats per minute and temperature of 99°F was lying on bed in agony. His abdomen had marked tenderness and guarding in the right iliac region. Leucocyte count was 15600 per mm³ and urinalysis was normal. Ultrasound abdomen showed minimal amount of fluid in the right iliac fossa. Provisional diagnosis of acute appendicitis was made; at exploration moderate amount of blood stained fluid was found in the peritoneal cavity. Appendix appeared normal. Further exploration revealed a congested omentum twisted around itself (Fig. 1). Appendectomy and omentectomy of the affected omentum were done. Post-operative recovery was uneventful.

**Figure:**
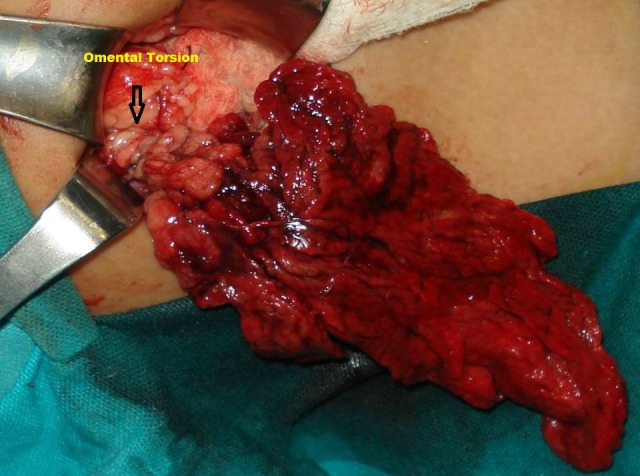
Figure 1:Omental torsion

**Case 2**

An 11-year-old girl presented with right lower quadrant pain for 3 days. It was associated with low grade fever for one day. On examination, pulse was 80 beats per minute and temperature 99°F. There was mild tenderness present in the right lower quadrant. Leucocyte count was 8600 per mm³ with normal urinalysis report. Ultrasound abdomen was reported normal. The next morning, her pain abdomen worsened with one episode of bilious vomiting. Her abdomen became markedly tender with guarding in the right iliac fossa. Upon exploration, small amount of serosanguineous fluid was found in the peritoneal cavity. Appendix was found to be normal. Further exploration revealed a congested omentum twisted around itself. Appendectomy and omentectomy of the affected omentum were done. Post-operative recovery was uneventful.

## DISCUSSION

Omental torsion is rare in children and out of more than 300 reported casesonly 15% occurred in children.[6]Omental torsion can be primary or secondary. In the absence of any associated intra-abdominal pathology, if a segment of omentum rotates around its own axis, it is a primary lesion.[7] Primary Omental torsion accounts for one-third of the cases. The etiology remains unclear but several factors have been implicated in the pathogenesis, such as anatomical malformations of the omentum, local variations in omental fat distribution as in obesity, vascular abnormality of omentum, trauma, and hyperperistalsis due to heavy meals and increased intra-abdominal pressure due to exercise or coughing. Obesity is considered to be an important predisposing factor and is present in almost 70% of patients.[5]Secondary torsion is more common and occurs as a result of underlying abdominal pathology (e.g. cysts, adhesions, hernia sacs) resulting in distal fixation point.[4]

Patients with omental torsion frequently presents with pain and tenderness in the right lower quadrant of abdomen, thus mimic acute appendicitis. It is very difficult to diagnose it preoperatively. Obesity, paucity of GI symptoms and relatively long duration of symptoms should raise the suspicion of primary omental torsion. Color doppler ultrasonography and computed tomography are useful diagnostic modalities. The presence of free serosanguineous fluid within the peritoneum cavity with normal appendix should raise the possibility of primary omental torsion. On CT scan a mass of fat density may be present showing whirling pattern of concentric streaks.[8]Management of omental torsion is relatively straight forward with resection of the involved omental segment. But some authors have suggested a conservative approach if the diagnosis of omental torsion is confirmed and other pathologies have been ruled out.[9]Surgical treatment results in a much faster recovery and pain alleviation. It also prevents possible sepsis and longer duration of hospital stay.[7]In conclusion, though very rare, omental torsion should be ruled out if normal appendix is found during surgery for acute appendicitis.

## Footnotes

**Source of Support:** Nil

**Conflict of Interest:** None declared

